# Development of a DualEmission Laser-Induced Fluorescence (DELIF) Method for Long-Term Temperature Measurements

**DOI:** 10.3390/s24227136

**Published:** 2024-11-06

**Authors:** Koji Toriyama, Shumpei Funatani, Shigeru Tada

**Affiliations:** 1 Graduate Faculty of Interdisciplinary Research, University of Yamanashi, 4-3-11 Takeda, Kofu 400-8511, Japan; sfunatani@yamanashi.ac.jp; 2 Department of Applied Physics, National Defense Academy, 1-10-20 Hashirimizu, Yokosuka 239-8686, Japan; stada@nda.ac.jp

**Keywords:** dual-emission laser-induced fluorescence method, long-term measurement, temperature resolution, excitation time, bandpass filter, high-speed monochrome camera, uncertainty analysis

## Abstract

The fluorescence intensity of fluorescent dyes typically employed in the dual-emission laser-induced fluorescence (DELIF) method gradually degrades as the excitation time increases, and the degradation rate depends on the type of fluorescent dye used. Therefore, the DELIF method is unsuitable for long-term temperature measurements. In this study, we focused on the fluorescence intensity ratio of a single fluorescent dye at two fluorescence wavelengths and developed a DELIF method for long-term temperature measurements based on this ratio. The fluorescence intensity characteristics of Fluorescein disodium and Rhodamine B, which are typically used in the DELIF method, in the temperature range of 10–60 °C were comprehensively investigated using two high-speed monochrome complementary metal-oxide semiconductor cameras and narrow bandpass filters. Interestingly, the ratio of the fluorescence intensity of each fluorescent dye at the peak emission wavelength of the fluorescence spectrum, λ, to the fluorescence intensity at wavelengths very close to the peak wavelength (λ ± 10 nm) was highly sensitive to temperature variations but not excitation time. Particularly, when Rhodamine B was used, the selection of the fluorescence intensity ratios at a wavelength combination of 589 and 600 nm enabled highly accurate temperature measurements with a temperature resolution of ≤0.042 °C. Moreover, the fluorescence intensity ratio varied negligibly throughout the excitation time of 180 min, with a measurement uncertainty (95% confidence interval) of 0.045 °C at 20 °C. The results demonstrate that the proposed DELIF method enables highly accurate long-term temperature measurements.

## 1. Introduction

Laser-induced fluorescence (LIF) is widely employed to measure the temperature distributions of liquids by correlating the fluorescent intensity of a fluorescent dye with the temperature [1,2,3]. Most studies have employed Rhodamine B, a red fluorescent dye, for LIF measurements owing to its wide fluorescence intensity variation with temperature of approximately 2%/°C at 27 °C [2,4,5,6]. However, the fluorescence intensity strongly depends on the incident light intensity. Therefore, the temperature distribution obtained through the LIF method inevitably includes some measurement errors owing to the non-uniform intensity distribution in the laser light sheet used as the light source.

Dual-emission LIF (DELIF), which uses two fluorescent dyes [7,8,9,10,11,12,13], is an alternative method that can address this issue as it eliminates the spatial fluctuations in laser intensity by considering the fluorescence intensity ratio of two fluorescent dyes. Moreover, the images of the two fluorescence intensity distributions can be obtained through inexpensive charge-coupled device (CCD) cameras. For example, Coppeta et al. [7] and Sakakibara and Adrian [8,9] used Fluorescein (green) and Rhodamine 110 (orange) as the secondary fluorescent dyes for Rhodamine B. Specifically, Sakakibara and Adrian [9] reported a random temperature measurement error of <0.17 K for the combination of Rhodamine B and 110. Subsequently, Sutton et al. [14] reported that Fluorescein 27, a green fluorescent dye, enhances the temperature resolution as it exhibited large fluorescent intensity variations with temperature (3.5%/°C) in their experiments. Funatani et al. [15] extended this method by adding a fluorescent dye to a mist to enable direct measurements of the gas temperature. They employed the DELIF method and obtained a gas temperature uncertainty interval of ±0.99 K with a tracking time of <1.0 ms. However, although the DELIF method is excellent for temperature distribution measurements, its major limitation is the requirement of two fluorescent dyes. Even if the dye concentration is constant, the fluorescence intensity deteriorates with excitation time (photobleaching) [16]. Furthermore, the deterioration rate of the fluorescence intensity with excitation time varies with the type of fluorescent dye used. Therefore, the conventional DELIF method is unsuitable for long-term temperature measurement. For example, Coolen et al. [17] reported that when Rhodamine B is irradiated with an Nd:YAG laser, the fluorescence intensity decreases with the excitation time, dropping to 97% after 600 s. In particular, when measuring the temperature distribution within a small, confined space, the issue of fluorescence photobleaching owing to the excitation time cannot be ignored because the object being measured is continuously irradiated by the laser light sheet. For example, in the case of transient temperature-field measurements of microfluidic channels and microdevices, which have become popular in recent years [18], continuous laser irradiation is suitable for real-time tracking of temperature measurements because of its large Fourier number. Saeki et al. [18] examined the relationship between the fluorescence intensity ratios of a single fluorescent dye at two different wavelengths and the temperature using a spectroscope. They focused on the fact that the fluorescence intensity of Rhodamine B strongly depends on temperature and exhibits opposite behaviors at low and high wavelengths, and investigated the relationship between the temperature and fluorescence intensity ratios at fluorescence wavelengths of 550 and 600 nm. Interestingly, they suggested that the DELIF method can be employed using only Rhodamine B. Similarly, Bruchhausen et al. [19] and Lavieille et al. [20] measured the liquid temperature using two wavelength bands of Rhodamine B emission. However, these studies did not evaluate the effects of excitation time on temperature measurement accuracy.

Therefore, this study investigated the effects of excitation time on the fluorescence intensity of dyes commonly employed in the DELIF method. To comprehensively evaluate the long-term temperature measurement capability of the DELIF method, two high-speed monochrome complementary metal-oxide semiconductor (CMOS) cameras with narrow bandpass filters were used to acquire the fluorescence intensities of two high-temperature-sensitive fluorescent dyes, Fluorescein disodium [21] and Rhodamine B, across two different wavelength ranges. Specifically, the fluorescence intensity variations of the same dye with excitation time were examined across different wavelength ranges.

Since the purpose of this study is to develop a temperature measurement method, the paper is organized as follows: Section 2 introduces the measurement principle, Section 3 quantitatively evaluates thermo-optical properties of fluorescent dyes using a photodiode sensor and a spectrometer, Section 4 evaluates the accuracy of the newly developed measurement method using two CMOS cameras, and Section 5 is the conclusion.

The results showed that a combination of Rhodamine B excitation wavelengths of 589 and 600 nm yielded the most favorable results and its fluorescence properties were negligibly affected by the excitation time. Additionally, the temperature uncertainty (95% confidence interval) under an excitation time of 180 min was 0.045 °C at 20 °C. Therefore, the proposed DELIF method not only enables accurate long-term measurement of two- and three-dimensional temperature fields but also accurate temperature history measurements with inexpensive optical instruments because it eliminates the need to compensate for the transient degradation of fluorescent dyes.

## 2. Basic Principle of the Proposed DELIF Method

The fluorescence energy of a temperature measurement target irradiated with a laser beam is calculated as follows [8]:

(1) $$I_{e}\left(T,x,y\right)=I_{0}\left(x,y\right)\cdot C\cdot \phi \left(T,x,y\right)\cdot \varepsilon ,$$

where T=T(x,y) is the temperature, $I_{0}=I_{0}\left(x,y\right)$ is the incident laser intensity, C is the solution concentration, $\phi =\phi \left(T,x,y\right)$ is the quantum efficiency, and ε is the absorption coefficient. Additionally, $\left(x,y\right)\;$ denotes the coordinates of the 2D image of the fluorescence energy. Because ϕ is a function of T, if $I_{0}$, C, and ε are known, T can be determined using $I_{e}$. The fluorescence intensity captured as an image, $I=I\left(T,x,y\right)$, using a camera is expressed as

(2) $$I\left(T,x,y\right)=I_{e}\left(T,x,y\right)\cdot t_{s}\cdot S\cdot L\left(x,y\right),$$

where $t_{s}$ is the camera exposure time, S is the sensitivity of the optical system, and $L=L\left(x,y\right)$ is a constant that depends only on the optical system structure. Equation (2) can be modified by substituting it with Equation (1) as follows:

(3) $$I\left(T,x,y\right)=I_{0}\left(x,y\right)\cdot C\cdot \phi \left(T,x,y\right)\cdot \varepsilon \cdot t_{s}\cdot S\cdot L\left(x,y\right).$$

It is difficult to obtain the temperature distribution, T(x,y), because $I_{0}$ and L on the right-hand side of Equation (3) depend on the image coordinates $\left(x,y\right)$ and are difficult to determine. The ratio of the fluorescence intensity distribution of two different fluorescent dyes with peak-emission wavelengths of $\lambda_{1}$ and $\lambda_{2}$ is expressed as

(4) $$\frac{I_{\lambda_{1}}\left(T,x,y\right)}{{\;I}_{\lambda_{2}}\left(T,x,y\right)\;}=\frac{I_{0}\left(x,y\right)\cdot C_{\lambda_{1}}\cdot \phi_{\lambda_{1}}\left(T,x,y\right)\cdot \varepsilon_{\lambda_{1}}\cdot t_{s}\cdot S_{\lambda_{1}}\cdot L\left(x,y\right)}{\;I_{0}\left(x,y\right)\cdot C_{\lambda_{2}}\cdot \phi_{\lambda_{2}}\left(T,x,y\right)\cdot \varepsilon_{\lambda_{2}}\cdot t_{s}\cdot S_{\lambda_{2}}\cdot L\left(x,y\right)\;}\;=\frac{C_{\lambda_{1}}\cdot \phi_{\lambda_{1}}\left(T,x,y\right)\cdot \varepsilon_{\lambda_{1}}\cdot S_{\lambda_{1}}}{{\;C}_{\lambda_{2}}\cdot \phi_{\lambda_{2}}\left(T,x,y\right)\cdot \varepsilon_{\lambda_{2}}\cdot S_{\lambda_{2}\;}},$$

where $\lambda_{1}$ and $\lambda_{2}$ indicate the values for the first and second fluorescent dyes, respectively. This equation indicates that the fluorescence intensity ratio, $I_{\lambda_{1}\;}/I_{\lambda_{2}}$, depends only on T, and an accurate $I_{\lambda_{1}\;}/I_{\lambda_{2}}$ measurements would allow measuring the temperature with high accuracy. Thus Equation (4) can be rewritten as follows:

(5) $$\frac{{\;I}_{\lambda_{1}\;}}{I_{\lambda_{2}}}\left(T\left(x,y\right)\right)=\frac{C_{\lambda_{1}}\cdot \varepsilon_{\lambda_{1}}\cdot S_{\lambda_{1}}}{{\;C}_{\lambda_{2}}\cdot \varepsilon_{\lambda_{2}}\cdot S_{\lambda_{2}}}\cdot \frac{{\;\phi }_{\lambda_{1}\;}}{\;\phi_{\lambda_{2}}\;}\left(T\left(x,y\right)\right).$$

The temperature distribution can be obtained as follows:

(6) $$T\left(x,y\right)=T_{c}\left(\;\frac{{\;I}_{\lambda_{1}\;}}{I_{\lambda_{2}}}\;\left(x,y\right)\right),$$

where $T_{c}$ is the inverse function of the fluorescence intensity $I_{\lambda_{1}\;}/I_{\lambda_{2}}$. This is the underlying principle of the proposed DELIF method.

However, long-term excitation degrades a fluorescent dye and causes its fluorescence intensity to vary, even at a constant temperature. This degradation is attributed to the loss in photoactivation potential of fluorescent molecules by the excitation light [22,23]. To incorporate this characteristic into the phenomenological model, the quantum efficiency (ϕ) and absorption coefficient (ε) on the right-hand side of Equation (4) can be considered a function of the excitation time, t; thus, Equation (4) can be rewritten as follows:

(7) $$\frac{I_{\lambda_{1}}\left(T,t,x,y\right)}{{\;I}_{\lambda_{2}}\left(T,t,x,y\right)\;}=\frac{I_{0}\left(x,y\right)\cdot C_{\lambda_{1}}\cdot \phi_{\lambda_{1}}\left(T,t,x,y\right)\cdot \varepsilon_{\lambda_{1}}\left(t\right)\cdot t_{s}\cdot S_{\lambda_{1}}\cdot L\left(x,y\right)}{{\;I}_{0}\left(x,y\right)\cdot C_{\lambda_{2}}\cdot \phi_{\lambda_{2}}\left(T,t,x,y\right)\cdot \varepsilon_{\lambda_{2}}\left(t\right)\cdot t_{s}\cdot S_{\lambda_{2}}\cdot L\left(x,y\right)\;}=\frac{C_{\lambda_{1}}\cdot \phi_{\lambda_{1}}\left(T,t,x,y\right)\cdot \varepsilon_{\lambda_{1}}\left(t\right)\cdot S_{\lambda_{1}}}{{\;C}_{\lambda_{2}}\cdot \phi_{\lambda_{2}}\left(T,t,x,y\right)\cdot \varepsilon_{\lambda_{2}}\left(t\right)\cdot S_{\lambda_{2}\;}}.$$

Because the progression of fluorescence intensity degradation varies based on the type of fluorescent dye employed, ϕ and ε also vary differently for different dyes. Therefore, the time-dependent parameters on the right-hand side of Equation (7) cannot be eliminated, and the fluorescence intensity ratio cannot be expressed as a function of T and t alone. This implies that it is challenging to perform long-term measurements using the DELIF method. When using only one fluorescent dye, the ratio of its fluorescence intensities at two different wavelengths, $\lambda_{1}$ and ${\lambda_{1}}^{\prime }$, can be defined as follows:

(8) $$\frac{I_{\lambda_{1}}\left(T,t,x,y\right)}{\;I_{{\lambda_{1}}^{\prime }}\left(T,t,x,y\right)\;}=\frac{C_{\lambda_{1}}\cdot \phi_{\lambda_{1}}\left(T,t,x,y\right)\cdot \varepsilon_{\lambda_{1}}\left(t\right)\cdot S_{\lambda_{1}}}{{\;C}_{{\lambda_{1}}^{\prime }}\cdot \phi_{{\lambda_{1}}^{\prime }}\left(T,t,x,y\right)\cdot \varepsilon_{{\lambda_{1}}^{\prime }}\left(t\right)\cdot S_{{\lambda_{1}}^{\prime }}\;}\;=\frac{\phi_{\lambda_{1}}\left(T,t,x,y\right)\cdot \varepsilon_{\lambda_{1}}\left(t\right)\cdot S_{\lambda_{1}}}{\;\phi_{{\lambda_{1}}^{\prime }}\left(T,t,x,y\right)\cdot \varepsilon_{{\lambda_{1}}^{\prime }}\left(t\right)\cdot S_{{\lambda_{1}}^{\prime }}\;}\;\;(∵C_{\lambda_{1}}={\;C}_{{\lambda_{1}}^{\prime }}).$$

Furthermore, because only one type of fluorescent dye is considered above, $\varepsilon_{\lambda_{1}}\left(t\right)$ and $\varepsilon_{{\lambda_{1}}^{\prime }}\left(t\right)$ are expected to exhibit similar changes over time. Thus, Equation (8) can be rewritten as follows:

(9) $$\frac{{\;I}_{\lambda_{1}\;}}{{\;I}_{{\lambda_{1}}^{\prime }\;}}\left(T\left(x,y\right),t\right)=\frac{S_{\lambda_{1}}}{\;S_{{\lambda_{1}}^{\prime }}\;}\;\frac{{\;\phi }_{\lambda_{1}\;}}{\;\phi_{{\lambda_{1}}^{\prime }}\;}\left(T\left(x,y\right),t\right).$$

If the fluorescence intensity ratio, $I_{\lambda_{1}\;}/I_{{\lambda_{1}}^{\prime }}$, does not depend on t, the proposed DELIF method can be used to measure the temperature over long periods. The temperature distribution can be obtained using the following equation, which does not include the time t as a variable:

(10) $$T\left(x,y\right)={T_{c}}^{\prime }\left(\;\frac{{\;I}_{\lambda_{1}\;}}{\;I_{{\lambda_{1}}^{\prime }\;}}\;\left(x,y\right)\right),$$

where ${T_{c}}^{\prime }$ is an inverse function of the ratio of the fluorescence intensity $I_{\lambda_{1}\;}/I_{{\lambda_{1}}^{\prime }}$. Another advantage of the proposed DELIF method is that because Equation (9) does not include the concentration of fluorescent dye C, as a measurement parameter, temperature measurement is possible even when the concentration varies over time.

## 3. Thermo-Optical Properties of Fluorescent Dyes

To clarify the thermo-optical properties of the fluorescent dyes, preliminary experiments were carried out on the relationship between the fluorescence intensity and the excitation time, and the relationship between the fluorescence intensity and the temperature.

### 3.1. Fluorescence Intensity Variation with Excitation Time

#### 3.1.1. Experimental Setups

**Figure 1:**
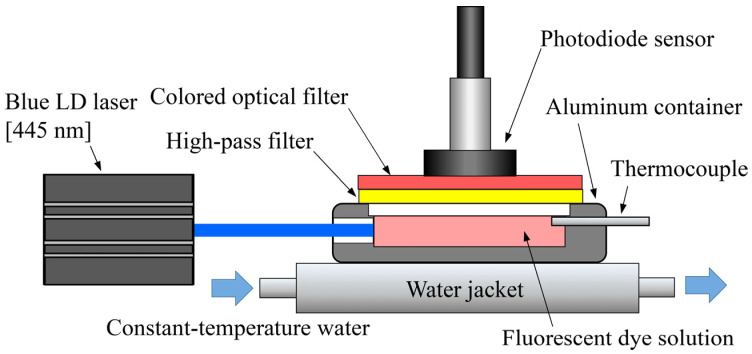
Experimental setup for fluorescence intensity measurements.

Figure 1 shows a schematic of the experimental setup used to evaluate the fluorescence intensity variations with excitation time. The fluorescent dye solution was placed in a container made of a cut-out aluminum block. The internal dimensions of the container were 5 (width) × 5 (depth) × 3 (height) mm and its upper portion contained a transparent 3 mm thick acrylic resin plate. Additionally, one of its side walls was made of transparent acrylic resin to allow laser light to enter it, whereas a K-type thermocouple was inserted on the opposite side to measure the solution temperature, which was controlled by a water jacket placed under the container. This jacket was composed of a 10 mm thick aluminum plate and its temperature was maintained by continuously circulating water heated to the target temperature. A blue semiconductor laser (LD; 445 nm, 1 W; Lasever, Inc., Ningbo, China) was used as the excitation light source to avoid increases in the solution temperature owing to incident light in the long-wavelength range. The fluorescence intensity of the dye solution was measured through a photodiode sensor (S228; Hamamatsu Photonics K.K., Shizuoka, Japan). A high-pass filter (SCF-50S-48Y, wavelength = 480 nm; Sigma Koki Corporation, Tokyo, Japan) was installed on the transparent plate placed on the top of the container to prevent the excitation light from directly entering the photodiode sensor. The fluorescent dye used in this experiment was Rhodamine B with a concentration of 0.6 ppm. A color optical filter (R60, wavelength = 640 nm; Hoya, Co., Ltd., Tokyo, Japan) was placed on the high-pass filter, and the fluorescence intensity obtained through the photodiode sensor was adjusted such that it was equal to that obtained through the CCD camera. All experiments were conducted in a dark room. Additionally, the temperature of the fluorescent dye solution was maintained at 20.0 °C, and measurements were performed every 3 min for 180 min. The data were analyzed by converting the acquired fluorescence intensities into 14-bit digital data using a photosensor amplifier (C9329; Hamamatsu Photonics K.K.).

#### 3.1.2. Results and Discussion

**Figure 2:**
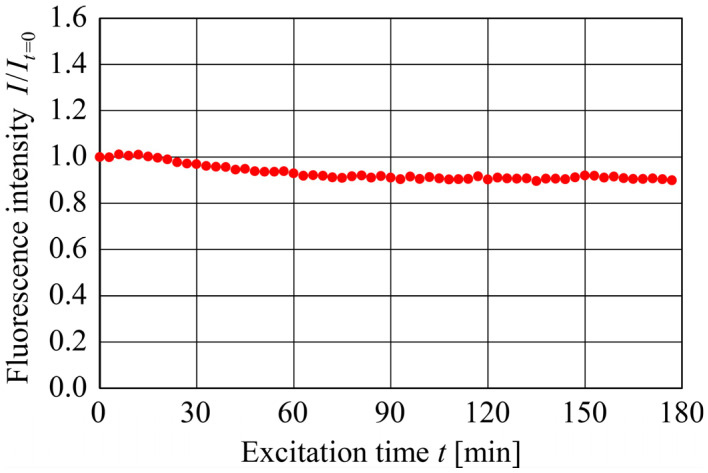
Fluorescence intensity variations of Rhodamine B, $I/I_{t=0}$, with excitation time t at T=20 °C.

Figure 2 shows the variations in the fluorescence intensity (*I*) of Rhodamine B with the excitation time, t, at a constant temperature of 20 °C. Note that I was normalized to the fluorescence intensity at t=0, i.e., $I_{t=0}$.

As can be seen, the fluorescence intensity gradually decreased as the excitation time increased, resulting in a reduction of approximately 10% after 180 min of excitation light exposure. This result implies that the standard DELIF method with conventional fluorescent dyes cannot be used for long-term temperature measurements.

### 3.2. Temperature-Dependent Photophysical Properties of Fluorescent Dyes

#### 3.2.1. Experimental Setups

**Figure 3:**
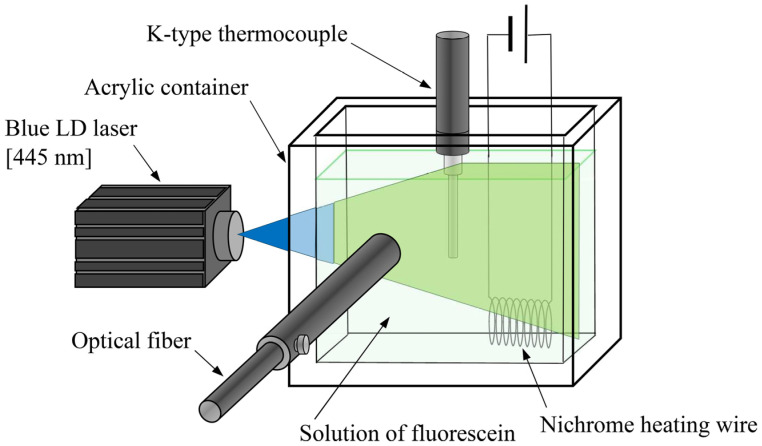
Experimental setup for fluorescence intensity variation measurements of Fluorescence disodium and Rhodamine B at temperature T.

Figure 3 shows a schematic of the experimental setup used to measure the fluorescence intensity variations with temperature using a spectrometer. The fluorescent dye solution was placed in a transparent acrylic resin container with internal dimensions of 7 (width) × 4 (depth) × 11 (height) cm and heated using a nichrome heating wire. The temperature was measured through the K-type thermocouple. The same blue semiconductor laser as that used in Section 3.1.1 (445 nm, 1 W; Lasever) was used as the excitation light for the fluorescence intensity measurements, and the laser sheet was irradiated through the transparent side of the container. The fluorescence intensity was measured using a spectrometer (Fastevert S-2431; Soma Optics, Ltd., Tokyo, Japan) connected through an optical fiber. All experiments were conducted in a dark room. Solutions comprising 0.04 ppm of Fluorescein disodium and Rhodamine B were used and subjected to 11 different temperatures, ranging from 10 to 60 °C, for the fluorescence intensity measurements.

#### 3.2.2. Results and Discussion

**Figure 4:**
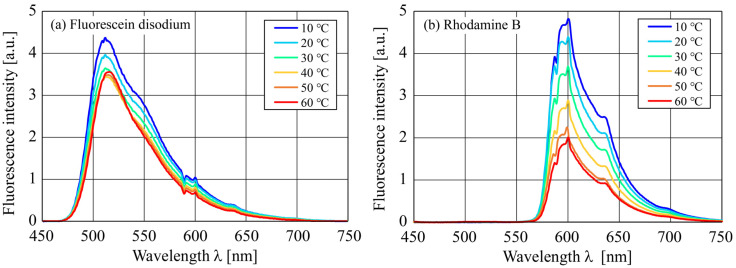
Relationship between the wavelength λ and fluorescence intensity at various temperatures T, for (**a**,**b**).

Figure 4 shows the relationship between the fluorescence wavelengths and intensities of the (a) Fluorescein disodium and (b) Rhodamine B solutions at various temperatures. Note that the fluorescence intensities on the ordinate are expressed in arbitrary units. As shown in Figure 4a, Fluorescein disodium exhibited a peak emission wavelength near 512 nm, and its fluorescence intensity decreased with temperature. However, at 50 and 60 °C, the peak emission started to increase, albeit slightly, and its wavelength shifted to longer wavelengths. This shift of the emission spectrum is called a bathochromic shift and is thought to be caused by the high pH sensitivity and concentration-dependent reabsorption of the dye in the shorter wavelength region, which acts as an internal filter for the emission of the dye, but the details of the mechanism are unknown [24]. Other than near the peak emission wavelength, the fluorescence intensity tended to decrease as the temperature increased. Furthermore, although Fluorescein disodium emits green fluorescence, it also emits a wide range of fluorescence up to a red wavelength of approximately 640 nm. Furthermore, as shown in Figure 4b, Rhodamine B exhibited a peak emission wavelength near 600 nm, and its fluorescence intensity decreased with temperature. Unlike the fluorescence spectrum of fluorescein disodium, no change was observed in the peak emission wavelength of Rhodamine B and its emission peak decreased monotonically as the temperature increased. Additionally, the fluorescence spectrum of Rhodamine B is widely distributed from approximately 560 nm to longer wavelengths. Particularly, in the wavelength range close to that of the peak emission, its fluorescence intensity changed significantly with the temperature. Therefore, by considering the fluorescence intensity ratio at the peak emission wavelength to that at nearby wavelengths, the value can be mapped one-to-one to the temperature with high accuracy. Thus, the solution temperature can be obtained with high accuracy using a single fluorescent dye.

**Figure 5:**
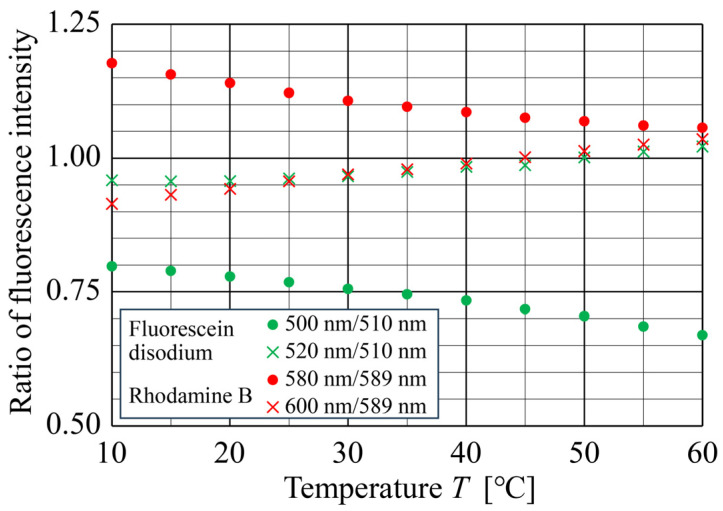
Relationship between the temperature and fluorescence intensity ratios of Fluorescein disodium for wavelength combinations of 500/510 nm and 520/510 nm and of Rhodamine B for wavelength combinations of 580/589 nm and 600/589 nm obtained using a spectrometer.

Figure 5 shows the temperature dependence of the fluorescence intensity ratios of Fluorescein disodium and Rhodamine B for various fluorescence–wavelength combinations from 10 to 60 °C. Specifically, we selected the peak emission wavelengths of 510 and 510 ± 10 nm (520 and 500 nm) for Fluorescein disodium and of 589 and 589 ± 10 nm (600 nm and 580 nm) for Rhodamine B for the experiment. Additionally, to calculate the fluorescence intensity ratio, we assumed that the fluorescence intensity at wavelength λ is $I_{\lambda }$. For example, “500 nm/510 nm” in the legend of Figure 5 indicates a fluorescence intensity ratio of $I_{500nm}/I_{510nm}$. As the temperature increased, the fluorescence intensity ratios of both fluorescent dyes decreased monotonically for combinations of peak emission wavelengths and those shorter than them. Conversely, for combinations of peak emission wavelengths and those longer than them, the fluorescence intensity ratios increased monotonically as the temperature increased. Therefore, in the temperature range of 10–60 °C, a two-way one-to-one mapping between the fluorescence intensity ratio and temperature was achieved, confirming the feasibility of measuring solution temperature using only a single fluorescent dye.

## 4. Temperature Measurement Capability of the Proposed DELIF Method

### 4.1. Experimental Setups

**Figure 6:**
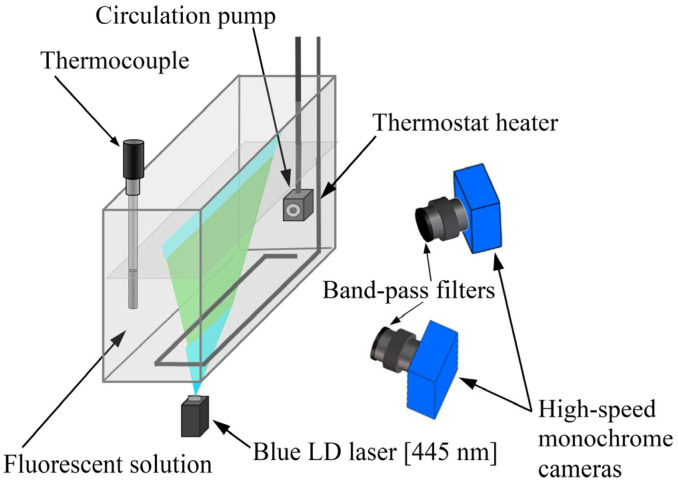
Experimental setup for the DELIF method with one dye. Two cameras and bandpass filters were used in this experiment.

Figure 6 shows a schematic of the experimental setup employed for the DELIF method using one fluorescent dye. Two solutions, one with 0.1 ppm of Fluorescein disodium and the other with 0.1 ppm of Rhodamine B, were prepared. Each solution was tested separately by placing it in a glass container with internal dimensions of 60 (width) × 350 (depth) × 150 cm (height). Subsequently, it was heated using a thermostat heater and stirred using a circulation pump to maintain a uniform temperature, which was measured using the K-type thermocouple. The same blue semiconductor laser used in the previous experiments was used as the excitation light for the fluorescence intensity measurements by irradiating the laser sheet from the bottom of the glass container. The reason for placing the light source below the container was due to the arrangement of the equipment that made up the experimental apparatus. The fluorescence intensity distribution was captured using two high-speed monochrome CMOS cameras (CP70-1-M-1000, 12-bit; Optronis GmbH, Kehl, Germany) equipped with bandpass filters (Edmund Optics, Co., Ltd., Barrington, NJ, USA). The cameras were positioned according to the Scheimpflug principle [25,26], which is an arrangement with a special geometric relationship among the focus-, lens-, and image-plane orientation of an optical system and enables perfect focus on a flat subject that is not parallel to the image plane. Among the bandpass filters attached to the camera, those with center wavelengths of 500, 510, and 520 nm were used for Fluorescein disodium measurements, whereas those with center wavelengths of 580, 589, and 600 nm were used for Rhodamine B measurements. The bandwidths (full width at half maximum) of all bandpass filters were set to 10 nm. The exposure time of the cameras was determined through preliminary experiments to ensure that sufficient grayscale images could be obtained for each wavelength. Table 1 lists the optimal camera exposure times for the six fluorescence wavelengths of Fluorescein disodium and Rhodamine B described above. All experiments were conducted in a dark room, and the fluorescence intensity was measured every 2 °C from 10 to 60 °C. Additionally, to mitigate measurement noise, the value of each pixel was averaged over 10 images, and the average value of the 10 × 10 pixels around the center of the acquired image was defined as the fluorescence intensity.

### 4.2. Results and Discussion

#### 4.2.1. Temperature Dependence of the Fluorescence Intensity Ratios

**Figure 7:**
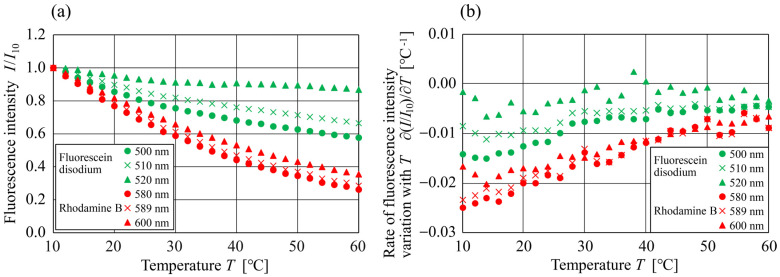
(**a**) Relationship between the temperature and fluorescence intensity and, (**b**) Relationship between the temperature and rate of fluorescence intensity variation with temperature, at different emission wavelengths for Fluorescein disodium (λ  = 500, 510, and 520 nm) and Rhodamine B (λ = 580, 589, and 600 nm).

Figure 7 shows (a) the relationship between the temperature and fluorescence intensity I, and (b) the relationship between the temperature and the rate of fluorescence intensity variation with temperature ∂I/∂T, of Fluorescein disodium and Rhodamine B for various fluorescence wavelengths. The value of I was normalized to the fluorescence intensity at T=10 °C, i.e., $I_{10}$. The fluorescence intensity decreased at all wavelengths as the temperature increased; however, the rate of reduction differed among the wavelengths. For both fluorescent dyes, the value of the rate of fluorescence intensity tended to increase as the wavelength decreased and the temperature increased (Figure 7b). The results also demonstrate that the variations in the temperature dependence of fluorescence intensity with the fluorescence wavelength could be observed even with inexpensive CMOS monochrome cameras and bandpass filters.

**Figure 8:**
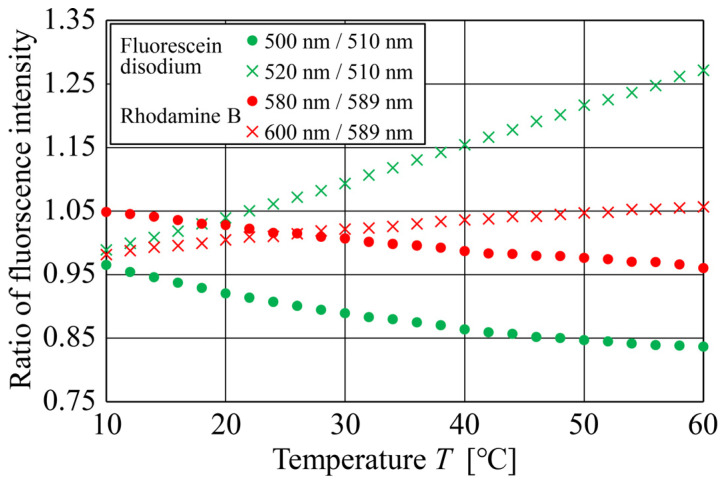
Relationship between the temperature and fluorescence intensity ratios of Fluorescein disodium for wavelength combinations of 500/510 nm and 520/510 nm and of Rhodamine B for wavelength combinations of 580/589 nm and 600/589 nm obtained using CMOS cameras and bandpass filters.

Figure 8 shows the relationship between the temperature and fluorescence intensity ratios for Fluorescein disodium and Rhodamine B under various combinations of fluorescence wavelengths. The fluorescence wavelengths of 510 and 589 nm are the peak emission wavelengths of Fluorescein disodium and Rhodamine B, respectively. Additionally, to calculate the fluorescence intensity ratio, we assumed that the fluorescence intensity at wavelength λ is $I_{\lambda }$. For example, “500/510” in the legend of Figure 8 represents the fluorescence intensity ratio of $I_{500\;nm}/I_{510\;nm}$. Similar to the results shown in Figure 5 obtained using a spectrometer, as the temperature increased, the fluorescence intensity ratios of both fluorescent dyes decreased monotonically for combinations of peak emission wavelengths and those shorter than them. Conversely, for combinations of peak emission wavelengths and those longer than them, the fluorescence intensity ratios increased monotonically as the temperature increased. Thus, the results obtained using CMOS cameras (Figure 8) exhibited a similar trend to those obtained using a spectrometer (Figure 5); however, the range of the fluorescence intensity ratios differed between them. Thus, bidirectional one-to-one mapping between the fluorescence intensity ratio and temperature was achieved, indicating that the proposed DELIF method using a single fluorescent dye can be used for temperature measurements.

#### 4.2.2. Temperature Resolution

**Figure 9:**
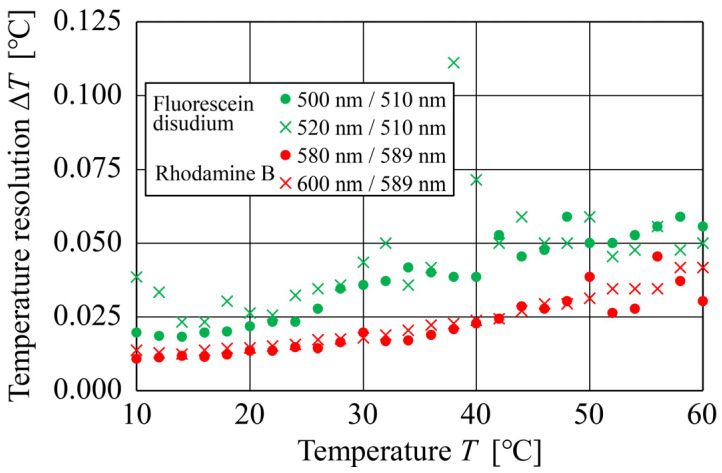
Relationships between the temperature and temperature resolutions of Fluorescein disodium for wavelength combinations of 500 and 510 nm and 520 and 510 nm, and of Rhodamine B for wavelength combinations of 580 and 589 nm and 600 and 589 nm.

Figure 9 shows the relationship between the temperature and temperature resolutions of Fluorescein disodium and Rhodamine B for the range of 10–60 °C obtained using the proposed DELIF method. Temperature resolution was defined as 1 °C divided by the number of identifiable grayscale gradations (increase or decrease in grayscale value per 1 °C). Because the fluorescence intensity decreases as the temperature increases, the temperature resolution also typically decreases as the temperature increases. Comparisons among the dyes showed that the temperature resolution of Fluorescein disodium was generally lower than that of Rhodamine B. Specifically, the temperature resolution of Fluorescein disodium was lower for the emission wavelength combination of 510 and 520 nm; however, in the range of 48–60 °C, the lowest resolution was obtained for the combination of 500 and 510 nm. Additionally, as the overall temperature resolution of Rhodamine B was higher than that of Fluorescein disodium, more accurate temperature measurement can be expected using Rhodamine B. In particular, the highest temperature resolution was obtained for the emission wavelength combination of 589 and 600 nm, with values of ≤0.042 °C in the range of 10–60 °C. The temperature resolution of 0.042 °C obtained in this study translates into a sensitivity of ~1.2%/K, which is equal to or higher than the sensitivity obtained by the conventional DELIF methods (~1.0–2.0%/K [12,27]).

#### 4.2.3. Dependence of Fluorescence Intensity Ratio on Excitation Time

To evaluate the variations in the fluorescence intensity ratio over long-term temperature measurements, a spectrometer was used to measure the fluorescence intensities of both Fluorescein disodium and Rhodamine B. The experimental setup was identical to that employed in Section 3, except that a bandpass filter was used instead of a color optical filter. Ten consecutive images were captured every 3 min for 180 min. The concentrations of Fluorescein disodium and Rhodamine B in the solutions were 0.4 and 0.6 ppm, respectively.

**Figure 10:**
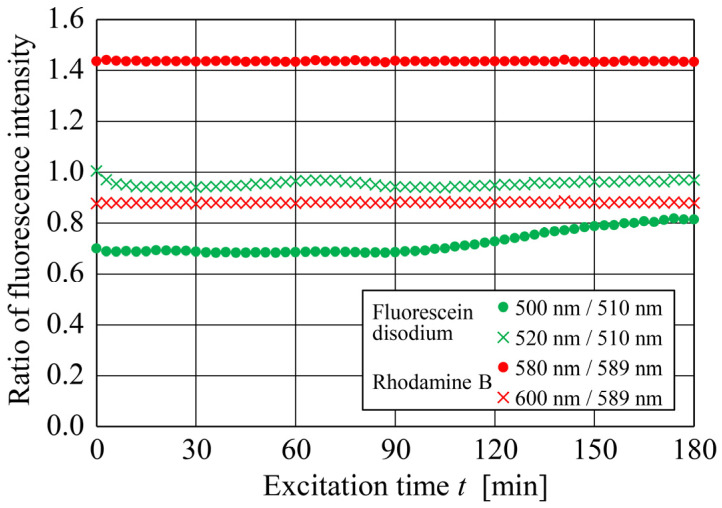
Relationships between the excitation time and fluorescence intensity ratios at 20 °C Fluorescein disodium for wavelength combinations of 500 and 510 nm and 520 and 510 nm, and of Rhodamine B for wavelength combinations of 580 and 589 nm and 600 and 589 nm.

Figure 10 shows the relationship between the excitation time and the fluorescence intensity ratio at 20 °C of both dyes for two emission wavelength combinations, wherein the time histories of their fluorescence intensity ratios exhibit distinct differences. The fluorescence intensity ratios of Fluorescein disodium exhibit complex variation with the excitation time, regardless of the emission wavelength combination. Specifically, its fluorescence intensity ratio for the emission wavelength combination of 510 and 520 nm decreased at the start of the excitation light exposure and then gradually started increasing with time. By contrast, that for the combination of 500 and 510 nm wavelengths remained almost constant for the first 90 min and then began increasing. However, the fluorescence intensity ratios of Rhodamine B remained nearly constant throughout the excitation time of 180 min for both emission wavelength combinations. Thus, the influence of the fluorescence intensity of the dye, which degrades with excitation time [17], on the temperature measurement was almost completely eliminated. To assess the degree of variation in the fluorescence intensity ratio with the excitation time, the uncertainty (U) under a 95% confidence interval over the 180 min temperature measurements was obtained as follows and evaluated:

(11) $$U=z\frac{\sigma }{\;\sqrt{\;n\;}\;}\;,$$

where z = 1.96 is the critical Z-score for the 95% confidence interval, σ is the unbiased standard deviation of the temperature measurements, and n is the sample size, which was 30 and 60 for the 90 and 180 min measurements, respectively. The uncertainty in the fluorescence intensity ratios of Fluorescein disodium for the emission wavelength combination of 500 and 510 nm up to 90 min was $U=4.61\times 10^{-3}$, whereas those for Rhodamine B for the wavelength combinations of 580 and 589 nm and 589 and 600 nm were considerably smaller at $U=2.60\times 10^{-3}$ and $U=2.23\times 10^{-3}$, respectively. However, the fluorescence intensity ratio of Fluorescein disodium increased after 90 min, indicating that it is unsuitable for long-term measurements. By contrast, that of Rhodamine B barely changed. Therefore, the uncertainty up to 180 min was only determined for Rhodamine B. The uncertainties (*U*) in the fluorescence intensity ratios of Rhodamine B up to 180 min for wavelength combinations of 580 and 589 nm and 589 and 600 nm were $2.38\times 10^{-3}$ and $2.25\times 10^{-3}$, respectively. By applying the temperature resolution to these values, the uncertainty in the measured temperature of Fluorescein disodium was T=0.092 °C (up to 90 min) and those for Rhodamine B were T=0.048 °C and T=0.045 °C (up to 180 min). These values for Rhodamine B are sufficiently accurate compared to those obtained using the conventional DELIF method [11,28,29]. Thus, the proposed DELIF method demonstrated its effectiveness for long-term temperature measurements across all cases.

## 5. Conclusions

This paper presented a new DELIF method that employs a single fluorescent dye for long-term temperature measurements. Two high-speed monochrome CMOS cameras with narrow bandpass filters were used to acquire the fluorescence intensities across two different wavelength ranges, and the ratio between them was analyzed to evaluate the possibility of long-term temperature measurements, which are difficult to achieve using the conventional DELIF method. Two fluorescent dyes, Fluorescein disodium and Rhodamine B, with different properties, were employed, and the temperature of each dye was continuously measured for 180 min. Subsequently, the stability of the measurements, temperature resolution, and uncertainty of the measured temperatures were investigated. The results demonstrated that temperature measurements in the range of 10–60 °C could be obtained with both fluorescent dyes. Additionally, the temperature resolution degraded as the temperature increased; however, the lowest degradation was observed with Rhodamine B, which exhibited a temperature resolution of 0.042 °C for an emission wavelength combination of 589 and 600 nm. Regarding the effect of excitation time, the fluorescence properties of Rhodamine B were hardly affected by prolonged excitation light exposure, with a temperature uncertainty (95% confidence interval) of ≤0.045 °C throughout the 180 min measurement. These results confirm that the proposed method is sufficiently accurate and reliable for long-term temperature measurements. Additionally, although this study only evaluated the grayscale values at the center of the image, the proposed method can be easily employed to measure the temperature distribution of confined liquids. Moreover, it not only enables accurate long-term measurements of both 2D and 3D temperature fields but also does not require the consideration of transient degradation compensation of the fluorescent dye. Therefore, it allows accurate long-term temperature history measurements through inexpensive optical instruments. However, it has a limitation in that it is not suitable for capturing rapid temperature changes. For example, the cameras required approximately 0.3 s to measure a single temperature field (10 images were captured with an exposure time of 0.03 s which was the optimal photographic condition as listed in Table 1). To further reduce the acquisition time, it is necessary to use a camera equipped with a CMOS sensor with better sensitivity characteristics and to review the configuration of the optical system, including the selection of filters and light sources. Future work will focus on transient 2D temperature measurements in confined spaces.

## Figures and Tables

**Table 1:** Camera exposure settings for each fluorescence wavelength.

Fluorescent Dye	λ [nm]	$t_{s}$ [×10^−2^ s]
Fluorescein disodium	500	2.4
	510	1.0
	520	6.6
Rhodamine B	580	3.0
	589	3.0
	600	4.9

## Data Availability

Data are contained within the article.
